# Relationship between pulse wave velocity progression and baseline heart rate and its change over 3.7 years of follow-up in hypertensive patients

**DOI:** 10.1097/HJH.0000000000004106

**Published:** 2025-07-24

**Authors:** Alessandro Maloberti, Chiara Tognola, Stefano Fumagalli, Ilaria Garofani, Michela Algeri, Atea Shkodra, Marco Bellomare, Marta Campana, Andrea Busti, Alfonso Riccio, Rita Facchetti, Guido Grassi, Michele Bombelli, Cristina Giannattasio

**Affiliations:** a School of Medicine and Surgery, University of Milano-Bicocca; b Cardiology 4, ASST GOM Niguarda, Milan; c Internal Medicine, Pio XI Hospital of Desio, ASST Brianza, Desio, Italy

**Keywords:** arterial hypertension, arterial stiffness, heart rate, pulse wave velocity, pulse wave velocity progression

## Abstract

**Objective:**

The role of resting heart rate (HR) and of its changes over time on the progression of pulse wave velocity (PWV) has not been extensively evaluated. The aim of this study was to investigate this relationship in a population of hypertensive patients in a longitudinal study.

**Methods:**

We enrolled 572 hypertensive outpatients aged 18–80, followed by the Hypertension Unit of St. Gerardo Hospital (Monza, Italy). Anamnestic, clinical and laboratory data, BP and PWV were assessed at baseline and after a median follow-up of 3.7 ± 0.5 years.

**Results:**

At baseline, mean age was 53.9 ± 12.7 years, SBP and DBP were 141.2 ± 17.8 and 86.5 ± 10.5 mmHg, HR was 65.6 ± 10.9 bpm and PWV was 8.6 ± 2.0 m/s. Despite an improvement in BP and therapies, at follow-up, a PWV increase (ΔPWV 0.5 ± 2.2 m/s). In patients with higher ΔHR, ΔPWV was significantly higher (0.82 ± 2.22 vs. 0.27 ± 2.25 m/s, *P* = 0.003). At multivariable stepwise regression, baseline HR showed a significant association with baseline PWV. However, neither HR nor ΔHR was significantly associated with ΔPWV. Same results were confirmed by mediation analysis.

**Conclusion:**

The main result of our study was that despite the presence of a significant association between baseline HR and baseline PWV, neither HR nor ΔHR was significantly associated with PWV progression (ΔPWV). On the contrary, baseline PWV and baseline BP (SBP, DBP, MBP and PP) and their changes during follow-up present significant association with an accelerated process of arterial stiffening.

## INTRODUCTION

An elevated heart rate (HR) is a marker of sympathetic hyperactivation [1–4] and has been associated to an increase in cardiovascular mortality and events in various cardiological conditions [5].

A possible explanation could be that high HRs are able to determine an earlier development and a faster progression of hypertension-mediated target organ damage. In fact, elevated HR has been associated with an increase in arterial stiffness (that is pulse wave velocity – PWV) in studies with pacing-induced tachycardia [6,7] and cross-sectional studies [8]. However, such kind of studies are not adequate to demonstrate a causal link between HR and PWV.

To the best of our knowledge, only three longitudinal studies were published on this topic all finding that high baseline HR values were associated to PWV progression [9–11]. However, only two of them collected second HR measurements demonstrating that an increase in HR (or persistently high values) during the follow-up was associated with a faster arterial stiffness progression [9,10]. Both these studies were conducted in general populations and using brachial–ankle PWV.

The main PWV determinants are age and hypertension [11]. In fact, the higher the blood pressure (BP) values the higher the PWV values, as elasticity of the vessels is impaired at higher diameter due to vessels stretching. However, the relationship is bidirectional, the higher the PWV the higher the BP. In fact, in the presence of arterial stiffening, the retrograde pressure wave adds to the anterograde one increasing BP values.

As evidenced by pacing-induced tachycardia studies, at high HR, the vessel is not able to return to its baseline dimension, due to a reduced diastolic time, before the next pulse wave arrives and this, obviously, determines an increase in the PWV measured at that time. Furthermore, high HR exposes the endothelium to low and oscillatory shear stress that determine local inflammation and oxidative stress impacting on arterial stiffness progression [12,13].

So, PWV does not only reflect the functional BP/HR-related vasoconstriction/relaxation but is related to the anatomical alterations of the intima and media layer that modulates vessels tone.

A further evaluation on the relationship between HR and PWV is of importance for different reasons: both HR and PWV have been related to subsequent cardiovascular outcomes but, as the two factors are strongly related, whether one could influence more the association over the other is not clearly defined; age is the main determinant of arterial stiffness but if HR, its changes over time, or an interaction between these two, is able to modify PWV age-related increase need to be further confirmed in longitudinal study; if PWV increase represents a cause or a consequence of HR increase is not completely elucidated by published studies; some methodological issues can influence PWV evaluation if measured at high HR.

The aim of our study was to investigate the relationship between resting HR and its change (ΔHR) during a follow-up of 3.7 years with baseline PWV and its changes (ΔPWV) in the same time span. Differently from the two previously cited longitudinal studies, we have used carotid–femoral PWV (the gold-standard arterial stiffness measurements) in hypertensive patients, a population particularly of interest due to its increased probability of PWV progression.

## METHODS

### Study population

From September 2006 to January 2010, we enrolled 572 outpatients (aged 18–80) affected by essential hypertension and treated with antihypertensive medications, followed by the Hypertension Unit of St. Gerardo Hospital (Monza, Italy). Those with atrial fibrillation, women who were pregnant, patients with acute cerebrovascular and cardiac events in the month before the study (defined as myocardial infarction, angina pectoris, heart failure, stroke, transient ischemic attacks and claudication) and with medical conditions that would modify the reliability of the study were excluded. Secondary hypertension was investigated by biochemical and instrumental assessment appropriated to the patient's presentation and conditions and those patients were excluded.

From July 2010 to December 2014, we performed the second visit, with a median follow-up time of 3.7 ± 0.5 years. Study protocol was approved by institutional ethics review committees of the institution involved (San Gerardo Hospital Ethical Committee, number 638-2006), and all participants provided informed written consent after being informed of its nature and purpose.

### Protocol

At first visit (baseline), we collected a comprehensive medical history and performed a complete physical examination on all patients. With the patient in the sitting position for at least 5 min and with the arm placed at heart level, blood pressure (BP) measurements were taken by a trained physician with a semiautomatic sphygmomanometer (OMRON M3, OMRON Healthcare Europe, Hoofddorp, The Netherlands). BP was measured twice, and the mean of the two measurements was used for the calculation.

Biochemical analyses were conducted using an automated Modular Analytics SWA system (Roche Diagnostics, Rotkreuz, Switzerland), employing enzymatic colorimetric assays for glucose (GOD-PAP method), total cholesterol (CHOD-PAP method), HDL cholesterol (third-generation enzymatic colorimetric method), triglycerides (GPO-PAP method), and LDL cholesterol estimated using the Friedewald equation. Creatinine levels were measured via the Jaffé kinetic colorimetric method, and glomerular filtration Rate (GFR) was calculated using the Cockcroft–Gault equation.

Height and weight were obtained to calculate the patients’ BMI. Waist circumference was assessed halfway between the lower ribs and the iliac crest. Diabetes was defined as a fasting plasma glucose higher than 126 mg/dl in two occasions or as the use of antidiabetic drugs. Patients were placed in the supine position and ECG (on which HR was measured), and PWV were evaluated after 10 min of rest.

At follow-up, patients’ medical history and physical examination were rechecked, a second set of two BP measurements was taken. BP and PWV were recorded through the same techniques and using the same protocol of baseline examination.

### Pulse wave velocity

Aortic stiffness was evaluated by PWV between the carotid and the femoral artery of the same side with the patient in the supine position. The pressure pulse waveforms were simultaneously obtained at the two arterial sites at the right side using an automatic device (Complior, Colson; Alam Medical, Paris, France) and their distance calculated by taking the distance between hip and neck via a rigid ruler. The values were corrected by a 0.8 factor according to the PWV measurement methods consensus documents, which states to use the subtraction method instead of the direct one when assessing the distance between the two measurement points [14]. Two measurements were obtained in each patient and the mean was used for the analysis.

In our laboratory, the intra-session within-operator and between-operator variability of PWV amounts, respectively, to a coefficient of variation of the mean value of 2 and to 4%, the corresponding value for the inter-session between-operator variability being 4%.

### Statistical analysis

Results are expressed as mean ± standard deviation for continuous normally distributed variables, median (I quartiles, III quartile) for continuous skewed variables and percentages for categorical data. ΔPWV and ΔHR represented the difference between the two variables measured at follow-up and at baseline, so their positive value means an increase of PWV or HR, while a negative one indicates their decrease.

Between-group differences at baseline and follow-up were assessed by paired Student *t* test, Mann–Whitney *U* test and *χ*^2^ test (or Fisher exact test when needed) for normally distributed, nonnormally distributed and categorical variables, respectively.

When patients were divided accordingly to median ΔHR (<9 vs. ≥9 bpm) unpaired Student *t*, Mann–Whitney test and *χ*^2^ test (or Fisher exact test when needed) were used for normally distributed, nonnormally distributed and categorical variables, respectively.

Age-based tertiles were calculated. The three tertile groups were compared using the *χ*^2^ test for proportions, the Kruskal–Wallis test and analysis of variance (ANOVA). Bonferroni correction was applied for post hoc pairwise comparisons.

Pearson's correlation analysis was used to examine associations between PWV and ΔPWV and other variables.

Four multiple linear regression models were used to assess which baseline variables were associated with PWV, with each model including a different BP (systolic, diastolic, mean and pulse pressure – SBP, DBP, MBP and PP, respectively) entered individually. A stepwise selection method was used to identify the relevant predictors and baseline HR, baseline BP (SBP, DBP, MBP or PP), sex, diabetes mellitus, glucose (log transform), β-blockers and diuretic were inserted as covariates.

The same set of models was applied to ΔPWV, including corresponding delta variables. As in the baseline analysis, one model was constructed for each BP and its changes (SBP, DBP, MBP, PP, ΔSBP, ΔDBP, ΔMBP and ΔPP). Similarly, a stepwise selection method was used to identify the relevant predictors and baseline and ΔHR, baseline and ΔBP (SBP, DBP, MBP or PP), baseline PWV, BMI (both baseline and follow-up), diabetes mellitus, α-blockers, diuretic and statins (the latter two both at baseline and follow-up) were inserted as covariates. Standardized estimates were showed in Tables 2 and 3, while the relative crude coefficients were showed in Supplementary Tables 3 and 4.

Finally, a mediation analysis was conducted to evaluate whether the predictive effect of HR (or ΔHR) on PWV (or ΔPWV) was mediated by BP (or ΔBP).

All data were analysed using IBM SPSS Software (version 26.0) and a *P* value of less than 0.05 was taken as the minimal level of statistical significance.

## RESULTS

### Population characteristics

Table 1 shows baseline and follow-up data of our hypertensive treated population. At baseline, mean age was 53.9 ± 12.7 years, and 56.7% of the patients were men and the average time since diagnosis of hypertension was 6.8 ± 7.9 years.

**TABLE 1:** Whole population data at baseline and follow-up

Variable	Baseline	Follow-up	*P* value
Number	572	572	–
Anamnestic and clinical data			
Age (years)	53.9 ± 12.7	57.9 ± 12.6	<0.001
Males (%)	56.7	–	–
Hypertension duration (years)	6.8 ± 7.9	–	–
Diabetes mellitus (%)	7.0	–	–
Smokers (%)	39.8	14.5	<0.001
BMI (kg/m^2^)	26.8 ± 4.0	26.9 ± 4.0	0.28
WC (cm)	93.8 ± 11.8	95.6 ± 12.3	<0.001
SBP (mmHg)	141.2 ± 17.8	132.6 ± 17.2	<0.001
DBP (mmHg)	86.5 ± 10.5	79.2 ± 10.5	<0.001
MBP (mmHg)	104.7 ± 11.2	97.0 ± 11.3	<0.001
PP (mmHg)	54.7 ± 15.4	53.5 ± 13.8	0.041
HR (bpm)	65.6 ± 10.9	74.5 ± 11.8	<0.001
ΔHR (bpm)	-	8.9 ± 12.4	-
Biochemical variables			
Total cholesterol (mg/dl)	203.4 ± 39.5	198.6 ± 41.2	0.615
HDL-cholesterol (mg/dl)	53.5 ± 12.0	54.4 ± 12.8	0.695
LDL-cholesterol (mg/dl)	129.7 ± 30.8	130.2 ± 34.5	0.904
Triglycerides (mg/dl)	105 (76–153)	104 (81–123)	0.957
Glucose (mg/dl)	85 (78–94)	98 (89–110)	0.004
Creatinine (mg/dl)	0.93 ± 0.21	0.94 ± 0.19	0.425
eGFR (mg/dl)	91.6 ± 19.7	90.5 ± 18.3	0.546
Therapies			
ACE inhibitors (%)	31.7	37.5	0.046
ARBs (%)	29.6	43.6	<0.001
CCB (%)	31.2	37.8	0.021
β-blockers (%)	23.5	29.8	0.019
α-blockers (%)	11.4	13.3	0.368
Diuretics (%)	30.8	36.3	0.06
Statins (%)	11.2	20.8	<0.001
Arterial stiffness			
PWV (m/s)	8.6 ± 2.0	9.1 ± 2.3	<0.001
ΔPWV (m/s)	-	0.5 ± 2.2	-

ACE, angiotensin-converting enzyme; ARB, angiotensin receptor blockers; CCB, calcium channel blockers; eGFR, estimated glomerular filtration rate; HDL, high-density lipoprotein; HR, heart rate; LDL, low-density lipoprotein; MBP, mean blood pressure; PP, pulse pressure; PWV, pulse wave velocity; WC, waist circumference.

During the 3.7 ± 0.5 years follow-up, an important proportion of patients stop smoking (from 39.8 to 14.5%, *P* < 0.001) and a slightly, but significant, increase was observed for waist circumference (from 93.8 ± 11.8 to 95.6 ± 12.3 cm, *P* < 0.001). BP values decreased (SBP: from 141.2 ± 17.8 to 132.6 ± 17.2 mmHg; DBP: from 86.5 ± 10.5 to 79.2 ± 10.5 mmHg; MBP: from 104.7 ± 11.2 to 97.0 ± 11.3 mmHg; PP: from 54.7 ± 15.4 to 53.5 ± 13.8 mmHg; *P* < 0.001 for all comparisons) with a significant improvement in BP control (from 33.4 to 60.8%). On the contrary, HR significantly increased (from 65.6 ± 10.9 to 74.5 ± 11.8 bpm, *P* < 0.001; ΔHR 8.9 ± 12.4 bpm).

Regarding laboratory data, only glucose had a significant variation during the follow-up with an increase from 85 (78–94) to 98 (89–110) mg/dl (*P* = 0.004).

The use of most classes of antihypertensive drugs increased: patients taking angiotensin-converting enzyme inhibitors rose from 31.7 to 37.5% (*P* = 0.046), angiotensin receptor blockers from 29.6 to 43.6% (*P* < 0.001), calcium channel blockers from 31.2 to 37.8% (*P* = 0.021) and β-blockers from 23.5 to 29.8% (*P* = 0.019). Furthermore, also lipid-lowering therapies increased from 11.2 to 20.8% (*P* < 0.001).

Despite significant improvement in BP and therapies, PWV increased significantly over that period (from 8.6 ± 2.0 to 9.1 ± 2.3 m/s, *P* < 0.001; ΔPWV 0.5 ± 2.2 m/s).

Supplementary Table 1 shows the population characteristics when divided accordingly to age tertiles (≤48 years, 49–60 years, >60 years). Older patients had longer hypertension duration, higher prevalence of diabetes mellitus, higher baseline and follow-up waist circumference values. Similarly, the baseline and follow-up SBP values were higher in the group of older patients, but they had also the higher SBP decrease during the follow-up. On the contrary, DBP values were lower in older patients both at baseline and follow-up without significant differences when Δ values were considered. As expected, MBP was similar between the three groups, while PP was higher in the third tertiles.

Also HR was lower in older patients both at baseline and follow-up evaluation with no difference in its Δ indicating that increase was similar irrespective of age groups.

PWV values were higher as the age increase both at baseline and follow-up, and the older group had a significantly higher increase.

Regarding biochemical variables, both the patients with an age older than 60 years and the one in the 49–60 years had higher baseline total cholesterol, triglycerides and glucose with lower GFR, while only the latter present significant difference at follow-up evaluation.

Finally, the use of all the drug classes was higher in the older group both at baseline and follow-up.

### Heart rate variation and pulse wave velocity progression

The population was divided into two groups using the median increase in ΔHR (<9 vs. ≥9 bpm) as cut-off (Supplementary Table 2). Patients in the group of ΔHR at least 9 bpm were more frequently smokers (45.4 vs. 22.6%, *P* = 0.007) and, despite similar baseline BMI and waist circumference values they showed higher follow-up values (BMI: 27.3 ± 4.1 vs. 26.5 ± 3.8, *P* = 0.016; waist circumference: 96.7 ± 12.3 vs. 94.5 ± 12.0, *P* = 0.028).

**FIGURE 1:**
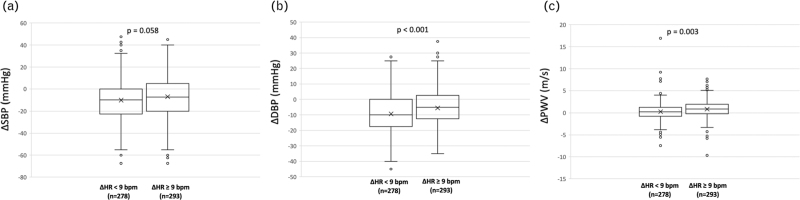
Comparison between the characteristics of the enrolled patients when divided accordingly to ΔHR median value: ΔSBP (panel a), ΔDBP (panel b) and ΔPWV (panel c).

There were no differences in baseline BP values as well as for ΔSBP (Fig. 1, panel a) and ΔPP while ΔDBP and ΔMBP were lower in the group with ΔHR ≥ 9 (ΔDBP: −5.4 ± 12.4 vs. −9.3 ± 12.3 mmHg, *P* < 0.001; Fig. 1, panel b; ΔMBP: −6.0 ± 13.4 vs. −9.6 ± 13.1, *P* = 0.001).

Baseline HR was significantly lower in the group ΔHR at least 9 bpm (61.8 ± 8.9 vs. 69.6 ± 11.3 bpm, *P* < 0.001).

While antihypertensive therapies at baseline were similarly distributed, at follow-up, patients with ΔHR at least 9 bpm had a lower prescription of β-blockers (25.6 vs. 34.2%, *P* = 0.028) and a higher prescription of calcium channel blockers (43.0 vs. 32.4%, *P* = 0.010) and alpha-blockers (17.1 vs. 9.4%, *P* = 0.007). Statins were more frequently prescribed at baseline in patients with ΔHR at least 9 bpm (14.2 vs. 7.9%, *P* = 0.017) but the difference was not significant at follow-up.

Regarding biochemical variables, despite similar baseline values, creatinine was higher at follow-up in patients with ΔHR at least 9 bpm (1.05 ± 0.17 vs. 0.86 ± 0.18, *P* = 0.028).

Finally, patients with ΔHR at least 9 were characterized by a lower baseline PWV (8.3 ± 1.8 vs. 8.9 ± 2.2 m/s, *P* = 0.001), but with a ΔPWV significantly higher (0.82 ± 2.22 vs. 0.27 ± 2.25 m/s, *P* = 0.003; Fig. 1, panel c).

### Multivariable regression models and mediator analyses

Multivariable stepwise regression models with baseline PWV as the dependent variable and baseline HR, baseline BP (SBP, DBP, MBP or PP), sex, diabetes mellitus, glucose (log transform), β-blockers and diuretic as covariates were done (Table 2, showing standardized coefficient). We found that baseline HR was a significant determinant of baseline PWV irrespective of the BP inserted into the model. Furthermore, sex, GFR, glucose (only for DBP model), diuretic use (only for MBP model) and β-blockers were significantly associated with baseline PWV values.

**TABLE 2:** Multivariable stepwise regression model (baseline pulse wave velocity)

Dependent variable: baseline PWV												

	SBP			DBP			MBP			PP		
Predictor	*β*	95% CI	*P* value	*β*	95% CI	*P* value	*β*	95% CI	*P* value	*β*	95% CI	*P* value
SBP (mmHg)	0.353	0.274, 0.433	<0.0001	–	–	–	–	–	–	–	–	–
DBP (mmHg)	–	–	–	0.120	0.033, 0.208	0.0073	–	–	–	–	–	–
MBP (mmHg)	–	–	–	–	–	–	0.266	0.183, 0.35	<0.0001	–	–	–
PP (mmHg)	–	–	–	–	–	–	–	–	–	0.335	0.256, 0.415	<0.0001
HR (bpm)	0.123	0.042, 0.205	0.0032	0.113	0.023, 0.205	0.0144	0.108	0.023, 0.194	0.0126	0.159	0.078, 0.242	0.0002
Sex (female)	−0.319	−0.48, −0.158	0.0001	−0.298	−0.475, −0.122	0.001	−0.311	−0.478, −0.145	0.0003	−0.347	−0.509, −0.187	<0.0001
β-blockers (yes)	0.317	0.122, 0.512	0.0015	0.345	0.13, 0.561)	0.0017	0.324	0.121, 0.529	0.0019	0.301	0.105, 0.498	0.0028
GFR (ml/min)	−0.202	−0.282, −0.123	<0.0001	−0.256	−0.343, −0.17	<0.0001	−0.218	−0.303, −0.135	<0.0001	−0.177	−0.258, −0.096	<0.0001
Glucose (mg/dl)	–	–	–	0.106	0.018, 0.195	0.0186	–	–	–	–	–	–
Diuretic (yes)	–	–	–	–	–	–	0.186	0.003, 0.37)	0.0458	–	–	–

Multivariable stepwise regression model with baseline PWV as the dependent variable and baseline HR, baseline BP (SBP, DBP, MBP or PP), sex, diabetes mellitus, glucose (log transform), β-blockers and diuretic as covariates. Standardized *β* shown. BP, blood pressure; GFR, Glomerular filtration rate; HR, heart rate; MBP, mean BP; PP, pulse pressure; PWV, pulse wave velocity.

**FIGURE 2:**
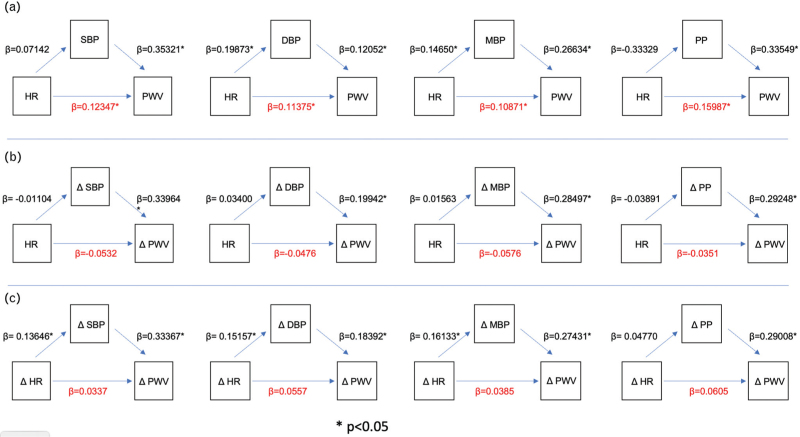
Path diagram of the mediation analysis of blood pressure values on the relationship between baseline heart rate and baseline pulse wave velocity (panel a), baseline HR and ΔPWV (panel b) ΔHR and ΔPWV (panel c). Results were adjusted for the same covariates used in the respective multivariable models.

Mediation analysis, adjusted for the same covariates of the previous models, showed similar results (Fig. 2, panel a). Baseline HR had significant direct effects on baseline PWV with also significant indirect effects through BP (SBP, DBP, MBP and PP) values.

Despite a significant univariate association between ΔPWV and baseline HR (*β* = −0.101, 95% CI = −0.182 to −0.018, *P* = 0.016) and ΔHR (*β* = 0.604, 95% CI 0.539–0.67, *P* < 0.001), at multivariable stepwise regression models no significant association were confirmed (Table 3, showing standardized coefficient). Models with ΔPWV as the dependent variable present as covariates baseline and ΔHR, baseline and ΔBP (SBP/ΔSBP, DBP/ΔDBP, MBP/ΔMBP or PP/ΔPP), baseline PWV, BMI (both baseline and follow-up), diabetes mellitus, α-blockers, diuretic and statins (the latter two both at baseline and follow-up). ΔPWV significant associated variables were baseline PWV values, baseline and ΔBP values (not for baseline DBP), diabetes mellitus, diuretic use at follow-up, baseline use of α-blockers (not for PP model) and statins use at follow-up (only for DBP model).

**TABLE 3:** Multivariable stepwise regression model (Δ pulse wave velocity)

Dependent variable: ΔPWV												

	SBP			DBP			MBP			PP		
				
Predictor	*β*	95% CI	*P* value	*β*	95% CI	*P* value	*β*	95%, CI	*P* value	*β*	95% CI	*P* value
SBP (mmHg)	0.247	0.154, 0.34)	<0.0001	–	–	–	–	–	–	–	–	–
DBP (mmHg)	–	–	–	0.023	−0.066, 0.113	0.606	–	–	–	–	–	–
MBP (mmHg)	–	–	–	–	–	–	0.118	0.029, 0.208	0.0096	–	–	–
PP (mmHg)	–	–	–	–	–	–	–	–	–	0.324	0.227, 0.422	<0.0001
ΔSBP (mmHg)	0.340	(0.255, 0.426)	<0.0001	–	–	–	–	–	–	–	–	–
ΔDBP (mmHg)	–	–	–	0.196	0.107, 0.287	<0.0001	–	–	–	–	–	–
ΔMBP (mmHg)	–	–	–	–	–	–	0.283	0.196, 0.371	<0.0001	–	–	–
ΔPP (mmHg)	–	–	–	–	–	–	–	–	–	0.294	0.206, 0.383	<0.0001
Baseline PWV (m/s)	−0.492	(−0.569, −0.811)	<0.0001	−0.428	−0.501, −0.356	<0.0001	−0.436	−0.51, −0.362	<0.0001	−0.544	−0.622, −0.467	<0.0001
Diabetes mellitus (yes)	0.532	(0.254, 0.205)	0.0032	0.443	0.152, 0.735	0.0029	0.532	0.25, 0.816	0.0002	0.482	0.201, 0.764	0.0008
Follow-up diuretics (yes)	0.184	(0.036, 0.333)	0.015	0.208	0.056, 0.36	0.0073	0.214	0.064, 0.364	0.0051	0.178	0.029, 0.329	0.0194
Baseline α-blockers (yes)	0.254	(0.034, 0.476)	0.0241	0.276	0.049, 0.504	0.0174	0.288	0.064, 0.513	0.012	–	–	–
Follow-up statins (yes)	–	–	–	0.188	0.007, 0.371	0.0416	–	–	–	–	–	–

Multivariable stepwise regression model with ΔPWV as the dependent variable and baseline and ΔHR, baseline and ΔBP (SBP/ΔSBP, DBP/ΔDBP, MBP/ΔMBP or PP/ΔPP), baseline PWV, BMI (both baseline and follow-up), diabetes mellitus, α-blockers, diuretic and statins (the latter two both at baseline and follow-up) as covariates. Standardized β shown. BP, blood pressure; HR, heart rate; MBP, mean BP; PP, pulse pressure; PWV, pulse wave velocity.

Also in this case, mediation analysis, adjusted for the same covariates of the previous models, showed similar results. Nor baseline HR (Fig. 2, panel b) nor ΔHR (Fig. 2, panel c) had significant direct effects on ΔPWV with indirect effects (particularly for ΔPWV) through BP values.

Crude *β* estimates of multivariable models were showed in Supplementary Tables 3 and 4 for PWV and ΔPWV, respectively.

## DISCUSSION

The main result of our study was that, despite the presence of a significant association between baseline HR and baseline PWV, neither HR nor ΔHR were significantly associated with PWV progression (ΔPWV). On the contrary, baseline PWV and baseline BP (SBP, DBP, MBP and PP) and their changes during follow-up presents significant association with an accelerated process of arterial stiffening.

The hypothesis under the present analysis is related to the multiple mechanisms through which elevated HR could determine a progression in arterial stiffness. Firstly, as also highlighted by studies on stimulation-induced tachycardia [6,15–19], there is a measurement problem. Indeed, at high HR, the diastolic time is reduced, and the pressure inside the vessel is augmented due to the increased cardiac output. So, the vessel is not able to return to its baseline dimension before the next pulse wave arrives and it is also stretched by the increased internal pressure. These two conditions determine an increase in the PWV values when taken at the time of a high HR. To avoid this problem, patients were required to rest for at least 10 min before PWV was measured. However, in addition to these acute mechanisms, there are some chronic ones that can contribute to PWV progression. The persistently high HR determines vessels’ hemodynamic changes: increased BP values, due to increased cardiac output, which determine higher vessel mechanical load and stress; an oscillatory shear stress that is more frequent and less intense than the normal one [13]. These two conditions are able to increase local inflammation and oxidative stress and to directly damage the vessels [12], finally exerting in PWV progression. Furthermore, the alteration in vessel recoil, as described in acute situation, generate a vicious circle that further stiffened the arterial wall. Finally, HR is a marker of sympathetic activation that could further exert negative effects due to its vasoconstrictor activity [1–4].

However, our results argue against this hypothesis, confirming that baseline PWV values and BP values (as well as their changes during follow-up) are the most important variables associated with PWV progression.

Our study has two points of novelty: we used carotid–femoral PWV instead of brachial–ankle PWV, and our population is composed only of hypertensive patients.

Regarding the first point, several differences exist between the two methods, the most important one being the amount of nonaortic vessels included in the measurements. In fact, while nonaortic vessels (i.e. the carotid one) are also included in carotid–femoral PWV evaluation, the amount is significantly greater for brachial–ankle PWV. In this evaluation, a large part of medium-sized and resistive arteries is included in the assessment. Some researchers claim that evaluating a longer arterial length and including even resistive arteries should add information to the measurements. However, it could also be interpreted as a confounding factor, as the stiffening process is mainly located at elastic vessels level (i.e. the aorta) more than in muscular and resistive ones. Furthermore, the issue determined by the inclusion of carotid vessel into the carotid–femoral length measurements has been mathematically solved by multiplying the path length for a 0.8 correction factor. This factor resolves the problem determined by the contemporary travelling of the pulse wave from the aortic arch to the carotid and through the descending aorta to the femoral artery [20]. In fact, all the methodological consensuses on arterial stiffness propose carotid–femoral PWV as the gold standard measurement [21,22].

Among the two systems used to evaluate carotid–femoral PWV (SphygmoCor and Complior), some differences exist. The SphygmoCor device uses an arterial tonometer for recording pressure waveforms sequentially at the two measurement points, and the propagation time is measured with the foot-to-foot methods. On the opposite, with the Complior system, the two waveforms are recorded simultaneously using mechanotransducers, and timing is referenced to the point of maximum systolic upstroke. Due to these differences, the Complior system is more prone to significant PWV variation determined by HR changes [6,17].

On the opposite, one could speculate that the SphygmoCor system could be influenced by short-term HR changes due to the fact the pressure waveforms are sequentially evaluated; however, these were not found in clinical studies [23]. This problem less affects the brachial–artery PWV in which resistive arteries also are evaluated and on which diastolic time has lesser influence of local hemodynamic parameter.

In summary, brachial–ankle and carotid–femoral PWV measurements are not interchangeable; therefore, it is of interest to enlarge the results related to PWV and HR to the latter, which, as already mentioned, is considered the gold-standard for arterial stiffness evaluation.

Regarding the second point, the two previous studies are based on general population, while our results widen the relationship between HR and PWV also to hypertensive patients. This population have specific characteristics that make it of particular interest, such as a higher probability of developing arterial stiffness due to the higher BP values; the higher prevalence of cardiovascular and noncardiovascular comorbidities that further increase the risk of elevated PWV values and its progression; the antihypertensive therapies that can not only determine an improvement on arterial stiffness due to BP values reduction but also with specific properties particularly for agents that act on the renin–angiotensin system [24,25]; and the lipid-lowering therapies, that is more frequently used in hypertensive patients than in the general populations, and that is able to reduce PWV progression [26].

The two points regarding antihypertensive and lipid-lowering therapies are very important and have been taken into account by inserting these therapies into the multivariable models. In our study, the use of β-blockers is of extreme interest due to their double effects on BP and HR. In particular, their use increases from 23.5 to 29.8% during the follow-up time, although its increase is lower in the group with ΔHR ≥ 9 bpm.

In fact, β-blocker and diuretic (only for MBP model) use presents significant association with baseline PWV and follow-up diuretics, baseline α-blockers (not for PP model) and follow-up statins use (while only for DBP model) presents significant association with arterial stiffness progression. All these association were positive meaning that their use is associated with higher PWV values and a higher progression probability. These results further confirm the already known protective effects of renin–angiotensin system inhibitors [24,25] that was also recently found in the SPARTE trial (Strategy for Preventing cardiovascular and renal events based on ARTErial stiffness; NCT02617238) [27]. This trial was designed to evaluate a strategy of antihypertensive therapy intensification based on PWV values comparing it with a conventional therapy up-titration based on BP values. Although a reduction in cardiovascular events was not found (hazard ratio, 0.74, 95% CI 0.40–1.38, *P* = 0.35), patients randomized to a renin–angiotensin system inhibitor in combination with calcium channel blockers have a lower increase in PWV, confirming a protective effect of this combination on vascular aging.

Regarding β-blockers, they were found to exert a similar effect on PWV as that of drugs active on the renin–angiotensin system [28,29]. However, our study found the opposite results, but β-blockers are a heterogenous drug class, and the possibility exists that some molecules act differently from others, depending on some specific properties (i.e. nitric oxide release, such as for Nebivolol) or based on their β-receptor specificity. However, few studies have been published comparing different β-blockers. In one study, Nebivolol had the same effects on PWV than Atenolol, while there was a higher improvement in other hemodynamic markers (pulse pressure, augmentation index) that are associated to the arterial stiffening process [30]. In another study, atenolol vs. bisoprolol had the same effects on hemodynamic markers but PWV was not assessed [31].

So, the difference between our study and the previous published longitudinal ones (where a significant association was found between PWV progression and HR changes), could be explained by the differences in population selection criteria (hypertension vs. general population) and by the possible effects of antihypertensive and lipid-lowering therapies.

Two other points of our study deserves to be mentioned, that is, the influence of age and diabetes on PWV progression.

Regarding age, a specific subgroup analysis permits to confirm its importance in the arterial stiffness process. In fact, the older group (> 60 years) presented higher baseline and follow-up SBP with lower HR at both timelines. No difference was seen in ΔHR indicating that the increase during the follow-up was similar irrespective of age groups. On the opposite, older patients had higher SBP decrease. Despite this, they had higher PWV increase during the follow-up also starting from high baseline PWV.

Concerning diabetes mellitus, our data confirm its well known association with PWV values [32] and its progression [33]. Diabetes mellitus has multiple specific adverse consequences on the visco-elastic properties of the arterial wall that can explain this association such as a higher prevalence of obesity and the presence of endothelial dysfunction, insulin resistance, hyperinsulinaemia, advanced glycation end-products, low-grade inflammation and increased oxidative stress [34,35].

The last point to be discussed is relative to the increase in mean PWV despite the significant decrease in BP values and the increased fraction of well controlled hypertensive patients, together with the increase in antihypertensive and lipid-lowering therapies during the follow-up. This issue has been already discussed in other articles [36], and many reasons can determine this result, that is, the inadequate prevalence of patients that reached the BP target; the time needed to reach the BP target; the hypertension therapies and the time of their initiation; the hypertension duration; the length of follow-up and the aging of the population during the course of the study.

In fact, the higher the exposure (both in term of intensity and time) the higher the mechanical damage of the artery and the stiffness. Furthermore, the higher the stiffness the less probable its reduction or, anyway, the longer the time necessary for its reduction. So, in our study, the relevance and/or duration of BP reduction were probably insufficient to determine an improvement in PWV.

Our study had some limitations. Firstly, although electrocardiographic assessment of HR is a more valid measurement than the pulse evaluation, data from 24 h electrocardiographic assessment could give further information such as HR variability. Its relationship with PWV has been done in a cross-sectional study [37] but never in a longitudinal one. Secondly, while serial measurements of PWV could be an element of high accuracy in dynamic assessment, it could determine logistic and economic difficulties in large population. Intrinsically present in any study with serial measurements of a biological variable, the possibility of the ‘regression-to-the-mean’ phenomenon and its associated ‘regression dilution bias’ should be acknowledged. Moreover, the absence of a more complete assessment of autonomic function and impairment, limits our interpretation of the relationship between HR and PWV. Finally, in the multifactorial context linking PWV, BP and HR, it could be difficult to assess all parameters that can influence the relationships, and it is possible that some of them have not been evaluated (such as inflammation and endothelial function).

In conclusion, our study found that in treated hypertensive patients, there is a significant relationship between baseline resting HR and baseline PWV but not between baseline HR and its changes (ΔHR) and arterial stiffness progression (ΔPWV). On the contrary, BP values were confirmed as fundamental determinants of PWV progression.

## ACKNOWLEDGEMENTS

Funding: this work was supported by: European Community Seventh Framework Programme (FP7/2007-2013) Grant Agreement n° 278249; Italian MUR Dipartimenti di Eccellenza 2023-2027 project (l. 232/2016, art. 1, commi 314 – 337); and A. De Gasperis Cardiology and Cardiac Surgery Foundation.

### Conflicts of interest

There are no conflicts of interest.

## Supplementary Material

Supplemental Digital Content
