# Supplementary material for: Relationship between pulse wave velocity progression and baseline heart rate and its change over 3.7 years of follow-up in hypertensive patients
Source: J Hypertens. 2025 Jul 24;43(10):1686–94. doi: 10.1097/HJH.0000000000004106 (PMC12404624; doi:10.1097/HJH.0000000000004106)
Supplement: Supplemental Digital Content [file jhype-43-1686-s001.docx]

**SUPPLEMENTARY TABLE 1.** Comparison between the characteristics of the enrolled subjects when divided accordingly to age tertiles.

|  | **Age (years - baseline)** | | |  |
| --- | --- | --- | --- | --- |
| **Variable** | **≤48** | **49-60** | **>60** | **p-value** |
| Number | 197 | 197 | 178 |  |
| **Baseline** |  |  |  |  |
| **Anamnestic and clinical data** |  |  |  |  |
| Age (years) | 40.2±6.9 | 55.2±3.4 | 68.1±5.2 | - |
| Males (%) | 63.5 | 55.3 | 58.2 | 0.252 |
| Hypertension duration (years) | 3.1±4.5^* | 6.5±6.6* | 11.2±9.6 | **<0.001** |
| Diabetes Mellitus (%) | 1.5^* | 8.1 | 11.9 | **0.0004** |
| Smokers (%) | 33 | 44.2 | 42.3 | 0.054 |
| BMI (Kg/m2) | 26.6±4.3 | 27±4 | 26.8±3.7 | 0.485 |
| WC (cm) | 92.2±13* | 93.6±11 | 95.6±11.1 | **0.019** |
| SBP (mmHg) | 137.4±16.1* | 139.3±16.7* | 147.6±19.1 | **<0.001** |
| DBP (mmHg) | 89.5±10.9* | 86.1±10.3* | 83.6±9.3 | **<0.001** |
| MBP (mmHg) | 105±116. | 103±11.3 | 104.9±10.9 | 0.3454 |
| PP (mmHg) | 49.7±12.0^* | 53.2±12.9* | 64.0±16.7 | **<0.001** |
| HR (bpm) | 68.8±11.5^* | 65.2±9.5 | 62.8±10.1 | **<0.001** |
| **Biochemical variables** |  |  |  |  |
| Total cholesterol (mg/dl) | 191.2±32.4* | 203.3±34.8 | 199.7±35.6 | **0.003** |
| HDL-cholesterol (mg/dl) | 53.4±13.8 | 51.9±13.5 | 54.2±13.3 | 0.300 |
| LDL-cholesterol (mg/dl) | 115.9±29.3 | 123.4±33.6 | 121.5±31.3 | 0.085 |
| Triglycerides (mg/dl) | 91.5 (66-130)^* | 111.5 (77-165) | 109 (81-155) | **0.0003** |
| Glucose (mg/dl) | 82 (76-89)^* | 85.5 (79-96) | 89 (80-101) | **<0.001** |
| Creatinine (mg/dl) | 0.84±0.16* | 0.85±0.2* | 0.92±0.24 | **0.001** |
| eGFR (mL/min) | 99.8±16.2^* | 91.2±19.3* | 82.1±19.8 | **<0.001** |
| **Therapies** |  |  |  |  |
| ACE-inhibitors (%) | 17.3^* | 35 | 44.1 | **<0.001** |
| ARBs (%) | 22.3* | 27.9* | 39.5 | **0.001** |
| CCB (%) | 29.9 | 28.9 | 35 | **0.402** |
| β-blockers (%) | 13.2^* | 24.9 | 33.3 | **<0.001** |
| α-blockers (%) | 5.6* | 11.2 | 18.1 | **0.0007** |
| Diuretics (%) | 11.2^* | 35.5 | 47.5 | **<0.001** |
| Statins (%) | 4.1* | 10.2* | 20.3 | **<0.001** |
| **Arterial stiffness** |  |  |  |  |
| PWV (m/s) | 7.8±1.4^* | 8.3±1.9* | 9.7±2.2 | **<0.001** |
| **Follow up** |  |  |  |  |
| **Anamnestic and clinical data** |  |  |  |  |
| BMI (Kg/m^2^) | 26.8±4.3 | 27.2±3.9 | 26.7±3.6 | 0.457 |
| WC (cm) | 93.6±12.9* | 96±11.7 | 97.5±11.7 | **0.008** |
| SBP (mmHg) | 130±15* | 132.2±17.7 | 136±18.2 | **0.003** |
| DBP (mmHg) | 81.9±10.5* | 79.5±10.1* | 75.6±10 | **<0.001** |
| MBP (mmHg) | 98.0±11.2 | 97.1±11.6 | 95.7±11.2 | **0**.1635 |
| PP (mmHg) | 48.1±10.3^* | 52.7±13.1* | 60.4±15.2 | **<0.001** |
| HR (bpm) | 77±12.8* | 74.7±11.3* | 71.6±10.4 | **<0.001** |
| **Biochemical variables** |  |  |  |  |
| Total cholesterol (mg/dl) | 197.6±41 | 198.4±45.6 | 193.2±38.8 | 0.954 |
| HDL-cholesterol (mg/dl) | 49.4±12.6 | 50.6±14 | 71.9±46.7 | 0.142 |
| LDL-cholesterol (mg/dl) | 117±42 | 123.6±33.3 | 98.4±35 | 0.382 |
| Triglycerides (mg/dl) | 123 (98-174) | 108 (81-118) | 85.5 (68-115) | 0.150 |
| Glucose (mg/dl) | 95 (84.5-99.5) | 102.5 (90-110) | 101.5 (84.5-113) | 0.512 |
| Creatinine (mg/dl) | 0.88±0.13 | 0.93±0.22 | 0.91±0.2 | 0.872 |
| eGFR (mL/min) | 111.8±16.6^* | 90.3±17.3 | 84.4±10.3 | **0.005** |
| **Therapies** |  |  |  |  |
| ACE-inhibitors (%) | 35.5 | 38.6 | 38.4 | 0.784 |
| ARBs (%) | 31.5^* | 45.2 | 55.4 | **<0.001** |
| CCB (%) | 35 | 34 | 45.2 | 0.051 |
| β-blockers (%) | 22.3* | 28.9 | 39 | **0.002** |
| α-blockers (%) | 9.6* | 9.6* | 21.5 | **0.0006** |
| Diuretics (%) | 19.8^* | 38.1* | 52.5 | **<0.001** |
| Statins (%) | 9.1^* | 21.3* | 33.3 | **<0.001** |
| **Arterial stiffness** |  |  |  |  |
| PWV (m/s) | 8±1.5^* | 9±2.1* | 10.5±2.5 | **<0.001** |
| **Delta** |  |  |  |  |
| ΔHR (bpm) | 8.2±13.8 | 9.9±12.3 | 8.8±11.2 | 0.389 |
| ΔPWV (m/s) | 0.21±1.72* | 0.63±2.02 | 0.85±2.88 | **0.020** |
| ΔSBP (mmHg) | -7.4±17.7 | -7.1±19.0 | -11.7±21.1 | **0.037** |
| ΔDBP (mmHg) | -7.5±13.4 | -6.5±12.3 | -8.0±11.6 | 0.502 |
| ΔMBP (mmHg) | -7.5±13.7 | -6.7±13.1 | 9.2±13.2 | 0.1782 |
| ΔPP (mmHg) | 0.2±12.9* | -0.6±15 | -3.7±17.0 | **0.0319** |

^p<0.05 vs age 49-60 years;* p<0.05 vs age>60 years

BMI = Body Mass Index; WC = Waist Circumference; SBP = Systolic Blood Pressure; DBP = Diastolic Blood Pressure; MBP = Mean Blood Pressure; PP = Pulse Pressure; HR = Heart Rate; HDL = High Density Lipoprotein; LDL = Low Density Lipoprotein; eGFR = estimated Glomerular Filtration Rate; ACE = Angiotensin Converting Enzyme; ARBs = Angiotensin Receptor Blockers; CCB = Calcium Channel Blockers; PWV = Pulse Wave Velocity.

**SUPPLEMENTARY TABLE 2.** Comparison between the characteristics of the enrolled subjects when divided accordingly to ΔHR median value.

| **Variable** | **ΔHR < 9 bpm (n=278)** | **ΔHR ≥ 9 bpm (n=293)** | **P-value** |
| --- | --- | --- | --- |
| **Anamnestic and clinical data** | | | |
| Age (years) | 53.9 ± 13.4 | 54.2 ± 11.6 | 0.763 |
| Males (%) | 57.6 | 60.4 | 0.497 |
| Hypertension duration (years) | 6.6 ± 7.8 | 6.9 ± 8.0 | 0.725 |
| Diabetes Mellitus (%) | 7.9 | 6.1 | 0.417 |
| Smokers (%) | 22.6 | 45.4 | **0.007** |
| Baseline BMI (Kg/m^2^) | 26.6 ± 4.0 | 27.0 ± 4.0 | 0.353 |
| Follow-up BMI (Kg/m^2^) | 26.5 ± 3.8 | 27.3 ± 4.1 | **0.016** |
| Baseline WC (cm) | 93.5 ± 12.2 | 93.9 ± 11.4 | 0.677 |
| Follow-up WC (cm) | 94.5 ± 12.0 | 96.7 ± 12.3 | **0.028** |
| Baseline SBP (mmHg) | 141.2 ± 18.1 | 141.3 ± 17.6 | 0.966 |
| Baseline DBP (mmHg) | 86.5 ± 10.8 | 86.5 ± 10.2 | 0.941 |
| Baseline MBP (mmHg) | 104.7 ± 11.2 | 104.7 ± 11.3 | 0.981 |
| Baseline PP (mmHg) | 54.7 ± 16.7 | 54.8 ± 14.1 | 0.920 |
| ΔSBP (mmHg) | -10.2 ± 19.3 | -7.1 ± 19.2 | 0.058 |
| ΔDBP (mmHg) | -9.3 ± 12.3 | -5.4 ± 12.4 | **<0.001** |
| ΔMBP (mmHg) | -9.6 ± 13.1 | -6.0 ± 13.4 | **0.001** |
| ΔPP (mmHg) | -0.9 ± 15.7 | -1.7 ± 14.4 | 0.501 |
| Baseline HR (bpm) | 69.6 ± 11.3 | 61.8 ± 8.9 | **<0.001** |
| **Baseline biochemical variables** | | | |
| Creatinine (mg/dl) | 0.86 ± 0.20 | 0.87 ± 0.21 | 0.452 |
| Glucose (mg/dl) | 85 (78.5-92) | 85 (78-96) | 0.884 |
| Total cholesterol (mg/dl) | 197.9 ± 34.4 | 198.0 ± 34.8 | 0.982 |
| HDL-cholesterol (mg/dl) | 54.7 ± 14.1 | 51.8 ± 13.0 | **0.021** |
| LDL-cholesterol (mg/dl) | 118.7 ± 31.9 | 121.5 ± 31.2 | 0.345 |
| Triglycerides (mg/dl) | 100 (73-151) | 107 (78-153) | 0.306 |
| **Follow-up biochemical variables** | | | |
| Creatinine (mg/dl) | 0.86 ± 0.18 | 1.05 ± 0.17 | **0.016** |
| Glucose (mg/dl) | 101 (89-111) | 96 (88-100.5) | 0.237 |
| Total cholesterol (mg/dl) | 199.1 ± 45.6 | 190.2 ± 27.8 | 0.592 |
| HDL-cholesterol (mg/dl) | 58.1 ± 32.4 | 51.4 ± 11.3 | 0.553 |
| LDL-cholesterol (mg/dl) | 114.8 ± 41.9 | 113.5 ± 27.3 | 0.938 |
| Triglycerides (mg/dl) | 93 (75.5 120.5) | 106 (103-174) | 0.657 |
| **Baseline therapies** | | | |
| ACE-Inhibitors (%) | 29.9 | 33.4 | 0.369 |
| ARBs (%) | 29.1 | 30 | 0.855 |
| CCB (%) | 28.4 | 33.8 | 0.176 |
| β-blockers (%) | 21.6 | 25.3 | 0.324 |
| α-blockers (%) | 9.7 | 13 | 0.237 |
| Diuretics (%) | 30.6 | 31.1 | 0.928 |
| Statins (%) | 7.9 | 14.3 | **0.017** |
| **Follow-up therapies** | | | |
| ACE-Inhibitors (%) | 36.3 | 38.6 | 0.604 |
| ARBs (%) | 42.4 | 44.7 | 0.613 |
| β-blockers (%) | 34.2 | 25.6 | **0.028** |
| Calcium-antagonists (%) | 32.4 | 43.0 | **0.010** |
| α-blockers (%) | 9.4 | 17.1 | **0.007** |
| Diuretics (%) | 34.2 | 38.2 | 0.338 |
| Statins (%) | 21.6 | 20.1 | 0.681 |
| **Arterial stiffness** | | | |
| Baseline PWV (m/s) | 8.9 ± 2.2 | 8.3 ± 1.8 | **0.001** |
| ΔPWV (m/s) | 0.27 ± 2.25 | 0.82 ± 2.22 | **0.003** |

BMI = Body Mass Index; WC = Waist Circumference; SBP = Systolic Blood Pressure; DBP = Diastolic Blood Pressure; MBP = Mean Blood Pressure; PP = Pulse Pressure; HR = Heart Rate; BMI = Body Mass Index; WC = Waist Circumference; HDL = High Density Lipoprotein; LDL = Low Density Lipoprotein; ACE = Angiotensin Converting Enzyme; ARBs = Angiotensin Receptor Blockers; CCB = Calcium Channel Blockers; PWV = Pulse Wave Velocity.

**SUPPLEMENTARY TABLE 3.** Multivariable stepwise regression model with baseline PWV as the dependent variable and baseline HR, baseline BP (SBP, DBP, MBP or PP), sex, diabetes mellitus, glucose (log transform), β-blockers and diuretic as covariates. Standardized β showed.

| **Dependent variable: baseline PWV** | | | | | | | | | | | | | | | | |
| --- | --- | --- | --- | --- | --- | --- | --- | --- | --- | --- | --- | --- | --- | --- | --- | --- |
|  | **SBP** | | | | **DBP** | | | | **MBP** | | | | **PP** | | | |
| **Predictor** | **β** | **(95%** | **C.I.)** | **P-value** | **β** | **(95%** | **C.I.)** | **P-value** | **β** | **(95%** | **C.I.)** | **P-value** | **β** | **(95%** | **C.I.)** | **P-value** |
| **SBP (mmHg)** | 0.040 | (0.031 | 0.049) | <0.0001 | - | - | - | - | - | - | - | - | - | - | - | - |
| **DBP (mmHg)** | - | - | - | - | 0.023 | (0.006 | 0.04) | 0.0073 | - | - | - | - | - | - | - | - |
| **MBP (mmHg)** | - | - | - | - | - | - | - | - | 0.048 | (0.033 | 0.063) | <0.0001 | - | - | - | - |
| **PP (mmHg)** | - | - | - | - | - | - | - | - | - | - | - | - | 0.044 | (0.034 | 0.055) | <0.0001 |
| **HR (bpm)** | 0.023 | (0.008 | 0.038) | 0.0032 | 0.021 | (0.004 | 0.038) | 0.0144 | 0.020 | (0.004 | 0.036) | 0.0126 | 0.030 | (0.014 | 0.045) | 0.0002 |
| **Sex (female)** | -0.646 | (-0.972 | -0.321) | 0.0001 | -0.605 | (-0.963 | -0.247) | 0.001 | -0.631 | (-0.968 | -0.293) | 0.0003 | -0.705 | (-1.032 | -0.378) | <0.0001 |
| **β-blockers (yes)** | 0.642 | (0.247 | 1.038) | 0.0015 | 0.701 | (0.264 | 1.137) | 0.0017 | 0.658 | (0.244 | 1.072) | 0.0019 | 0.611 | (0.212 | 1.01) | 0.0028 |
| **GFR (mL/min)** | -0.021 | (-0.029 | -0.013) | <0.0001 | -0.026 | (-0.035 | -0.017) | <0.0001 | -0.022 | (-0.031 | -0.014) | <0.0001 | -0.018 | (-0.027 | -0.01) | <0.0001 |
| **Glucose (mg/dL)** | - | - | - | - | 1.095 | (0.184 | 2.007) | 0.0186 | - | - | - | - | - | - | - | - |
| **Diuretic (yes)** | - | - | - | - | - | - | - | - | 0.379 | (0.007 | 0.75) | 0.0458 | - | - | - | - |

PWV = Pulse Wave Velocity; BP = Blood Pressure; SBP = Systolic BP; DBP = Diastolic BP; MBP = Mean BP; PP = Pulse Pressure; HR = Heart Rate; GFR = Glomerular Filtration Rate.

**SUPPLEMENTARY TABLE 4.** Multivariable stepwise regression model with ΔPWV as the dependent variable and baseline and ΔHR, baseline and ΔBP (SBP/ΔSBP, DBP/ΔDBP, MBP/ΔMBP or PP/ΔPP), baseline PWV, BMI (both baseline and follow-up), diabetes mellitus, α-blockers, diuretic and statins (the latter two both at baseline and follow-up) as covariates. Crude coefficient (β and 95% CI) showed.

| **Dependent variable:** Δ**PWV** | | | | | | | | | | | | | | | | |
| --- | --- | --- | --- | --- | --- | --- | --- | --- | --- | --- | --- | --- | --- | --- | --- | --- |
|  | **SBP** | | | | **DBP** | | | | **MBP** | | | | **PP** | | | |
| **Predictor** | **β** | **(95%** | **C.I.)** | **P-value** | **β** | **(95%** | **C.I.)** | **P-value** | **β** | **(95%** | **C.I.)** | **P-value** | **β** | **(95%** | **C.I.)** | **P-value** |
| **SBP (mmHg)** | 0.031 | (0.019 | 0.043) | <0.0001 | - | - | - | - | - | - | - | - | - | - | - | - |
| **DBP (mmHg)** | - | - | - | - | 0.005 | (-0.014 | 0.024) | 0.606 | - | - | - | - | - | - | - | - |
| **MBP (mmHg)** | - | - | - | - | - | - | - | - | 0.118 | (0.029 | 0.208) | 0.0096 | - | - | - | - |
| **PP (mmHg)** | - | - | - | - | - | - | - | - | - | - | - | - | 0.047 | (0.033 | 0.062) | <0.0001 |
| **ΔSBP (mmHg)** | 0.040 | (0.03 | 0.05) | <0.0001 | - | - | - | - | - | - | - | - | - | - | - | - |
| **ΔDBP (mmHg)** | - | - | - | - | 0.035 | (0.019 | 0.052) | <0.0001 | - | - | - | - | - | - | - | - |
| **ΔMBP (mmHg)** | - | - | - | - | - | - | - | - | 0.284 | (0.196 | 0.371) | <0.0001 | - | - | - | - |
| **ΔPP (mmHg)** | - | - | - | - | - | - | - | - | - | - | - | - | 0.044 | (0.031 | 0.057) | <0.0001 |
| **Baseline PWV** | -0.547 | (-0.631 | -0.462) | <0.0001 | -0.475 | (-0.556 | -0.395) | <0.0001 | -0.436 | (-0.51 | -0.362) | <0.0001 | -0.604 | (-0.69 | -0.518) | <0.0001 |
| **Diabetes Mellitus (yes)** | 1.198 | (0.572 | 1.823) | 0.0002 | 0.998 | (0.342 | 1.653) | 0.0029 | 0.533 | (0.25 | 0.816) | 0.0002 | 1.084 | (0.451 | 1.718) | 0.0008 |
| **Follow-up diuretics (yes)** | 0.415 | (0.081 | 0.749) | 0.015 | 0.208 | 0.2080 | 0.2080 | 0.0073 | 0.214 | (0.064 | 0.364) | 0.0051 | 0.402 | (0.065 | 0.739) | 0.0194 |
| **Baseline α-blockers (yes)** | 0.572 | (0.075 | 1.069) | 0.0241 | 0.208 | 0.2080 | 0.2080 | 0.0174 | 0.288 | (0.064 | 0.513) | 0.012 | - | - | - | - |
| **Follow-up statins (yes)** | - | - | - | - | 0.208 | 0.2080 | 0.2080 | 0.0416 | - | - | - | - | - | - | - | - |

PWV = Pulse Wave Velocity; BP = Blood Pressure; SBP = Systolic BP; DBP = Diastolic BP; MBP = Mean BP; PP = Pulse Pressure; HR = Heart Rate; BMI = Body Mass Index.
